# Bilateral Diffuse Uveal Melanocytic Proliferation Masquerading as Refractory Subretinal Fluid Due to Peripapillary Pachychoroid Syndrome

**DOI:** 10.7759/cureus.102512

**Published:** 2026-01-28

**Authors:** Gen Nakao, Chikako Hara, Kohji Nishida

**Affiliations:** 1 Ophthalmology, The University of Osaka Graduate School of Medicine, Suita, JPN

**Keywords:** bilateral diffuse uveal melanocytic proliferation, peripapillary pachychoroid syndrome, : prostate cancer, refractory disease, subretinal fluid

## Abstract

We describe a case of peripapillary pachychoroid syndrome (PPS) that appeared resistant to conventional treatment and was subsequently diagnosed as bilateral diffuse uveal melanocytic proliferation (BDUMP) based on fundus autofluorescence (FAF) images. A 79-year-old man presented with vision loss in both eyes and was initially diagnosed with PPS based on optical coherence tomography, fluorescein angiography, and indocyanine green angiography findings. The patient underwent photodynamic therapy in both eyes, photocoagulation in the left eye, and 2 mg aflibercept injections in both eyes. Although he responded to the initial treatments, the response gradually diminished with subsequent injections. Due to the progression of his cataracts, the patient underwent cataract removal surgery. He was later diagnosed with BDUMP based on subsequent FAF images, which revealed characteristic ocular fundus findings known as giraffe-like patterns. Subsequent systemic evaluation revealed prostate cancer. BDUMP can masquerade as PPS or neovascular age-related macular degeneration (n-AMD), resulting in delayed diagnosis and treatment, and should be considered in the differential diagnosis of cases where PPS or n-AMD is refractory to conventional treatment, even in the absence of a known malignancy.

## Introduction

Bilateral diffuse uveal melanocytic proliferation (BDUMP), first described by Machemer [1] in 1966, is an extremely rare, paraneoplastic intraocular disease characterized by multiple elevated and pigmented uveal lesions, diffuse thickening of the uveal tract, and rapidly progressive cataract(s) [2]. Ocular fundus findings known as giraffe-like patterns have recently been recognized on autofluorescence (FAF) as a characteristic feature in the diagnosis of BDUMP [2,3], which usually occurs in patients with various types of cancer, specifically ovarian cancer in women and lung cancer in men [3]. In nearly half of the reported cases, malignancies were discovered at the same time or shortly after the onset of BDUMP [3], a delay which contributes to the generally low life expectancy of these patients.

Several reports have highlighted that early stages of BDUMP may masquerade as neovascular age-related macular degeneration (n-AMD) [4-6] or central serous chorioretinopathy (CSC) [7], which may be one reason for delayed diagnosis and treatment. Peripapillary pachychoroid syndrome (PPS) is a pachychoroid spectrum disorder characterized by pachychoroid features predominantly localized around the optic nerve head, often associated with peripapillary and/or macular subretinal fluid (SRF). PPS may share clinical and imaging features with other pachychoroid-related disorders, which can make differentiation challenging in certain clinical settings. We describe herein a case of subretinal fluid (SRF) due to peripapillary PPS with a poor response to conventional treatment, in which the clinical features of BDUMP were observed, including giraffe-like patterns on AF images, leading to a diagnosis of BDUMP as well as the subsequent discovery of prostate cancer.

## Case presentation

**Figure 1:**
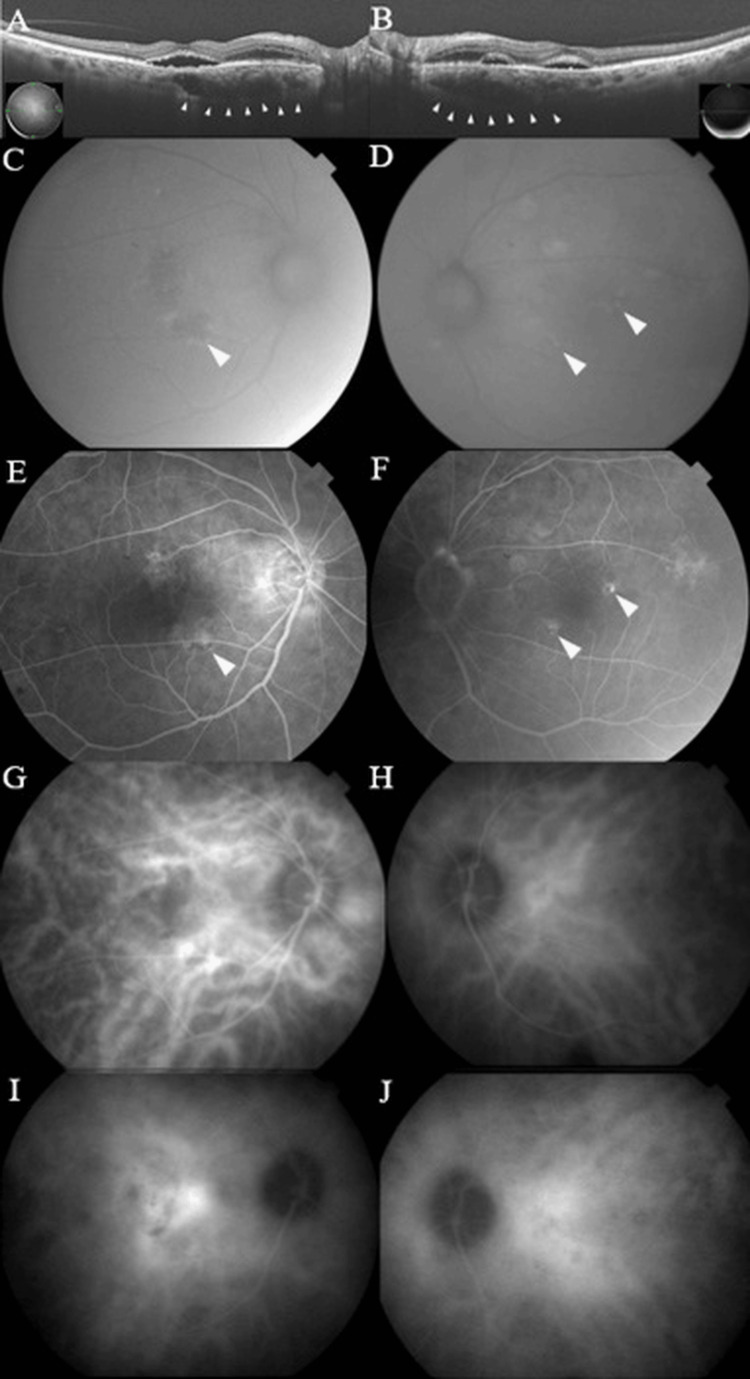
The examination at the first visit Swept-source optical coherence tomography (SS-OCT) revealed subretinal fluid (SRF), retinal edema from the papilla to the macula, and dilated choroidal vessels in both eyes. The choroidal thickness at the fovea and 1,500 µm nasal to the fovea was 357 µm and 539 µm in the right eye, and 414 µm and 504 µm in the left eye, respectively. (A,B). Fundus autofluorescence (FAF) imaging showed partial hyper- and hypofluorescent lesions in both eyes (C,D). Fluorescein angiography (FA) showed several areas of late phase pinpoint leakage in both eyes (E,F). Indocyanine green angiography (ICGA) showed early stage choroidal vasodilatation in both eyes (G,H) as well as middle-to-late stage hyperpermeability of the choroidal vessels in both eyes (I,J).

A 79-year-old Asian man who had previously been treated for glaucoma with ophthalmic drops presented with decreased vision in both eyes. He had also taken 10 mg of prednisolone for rheumatoid arthritis and immunoglobulin A nephropathy. His best-corrected visual acuity (BCVA) was 0.9 in the right eye and 1.0 in the left eye, and an anterior segment examination revealed clear corneas and anterior chambers, as well as mild cataracts bilaterally. Optical coherence tomography (OCT) revealed SRF, retinal edema from the optic disc to the macula, and dilated choroidal vessels in both eyes (Figure 1A,B). The choroidal thickness at the fovea and 1,500 µm nasal to the fovea was 357 µm and 539 µm in the right eye, and 414 µm and 504 µm in the left eye, respectively. In both eyes, the choroid was thicker on the nasal side than on the fovea. FAF images showed partial hyper- and hypofluorescent lesions (Figure 1C,D). Fluorescein angiography (FA) showed some hyperfluorescent spots in the late phases of both eyes (Figure 1E,F) and indocyanine green angiography (ICGA) showed vascular hyperpermeability between the optic disc and macula (Figure 1G-J).

Based on the examination results, the patient was diagnosed with SRF due to PPS. Reduced-fluence photodynamic therapy (RF-PDT) was administered in both eyes and focal photocoagulation (PC) was administered at the leakage point in the left eye. While the SRF had resolved in the right eye two months post-RF-PDT, it persisted in the left eye. Additional focal PC was administered in the left eye; however, the treatment was not successful. Intravitreal aflibercept (IVA; Eylea; Bayer, Basel, Switzerland) was subsequently administered in the left eye, and a complete resolution of the SRF was transiently observed. SRF recurred in both eyes; therefore, additional IVA was administered, after which the SRF resolved.

**Figure 2:**
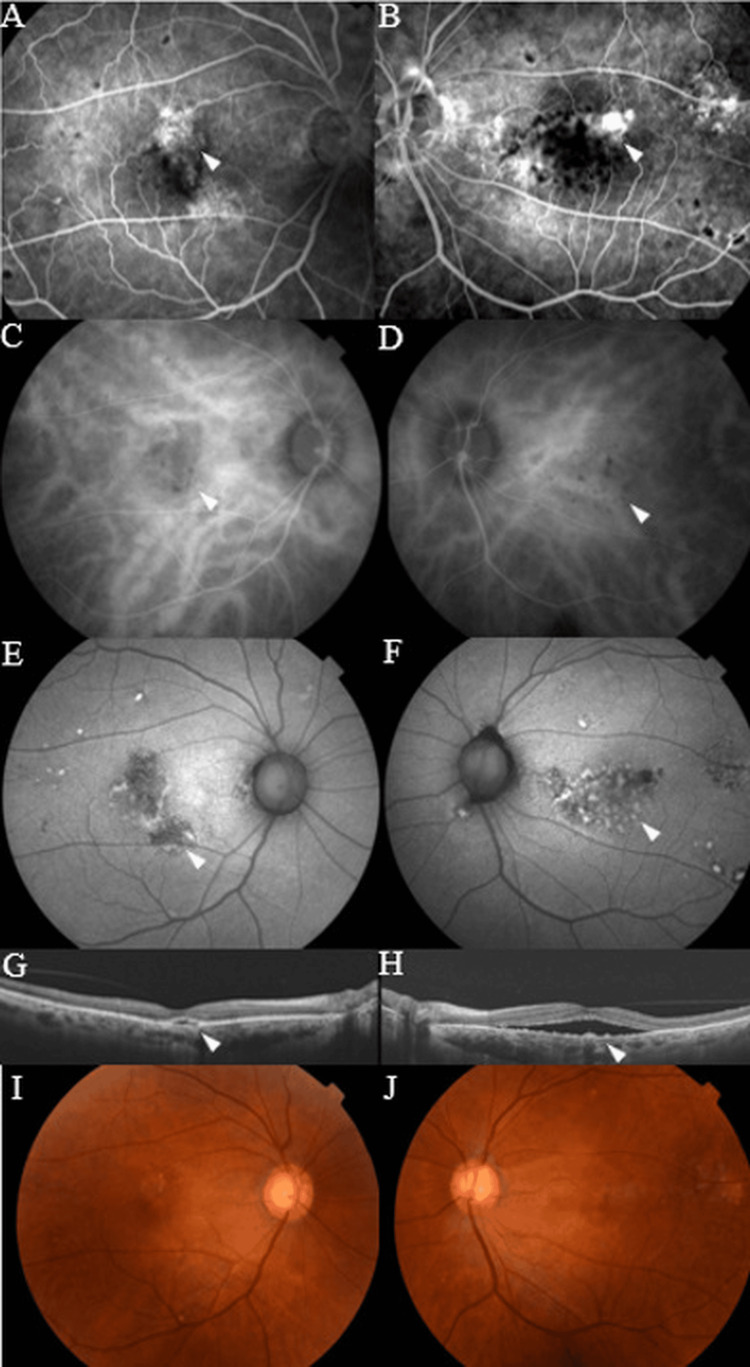
The examination when diagnosed with BDUMP Fluorescein angiography (FA) (A,B) and indocyanine green angiography (ICGA) (C,D) showed clear punctate hypofluorescent spots in both eyes, while fundus autofluorescence (FAF) imaging showed characteristic giraffe-like patterns in both eyes (E,F). Swept-source optical coherence tomography (SS-OCT) revealed well-depicted SRF with irregular retinal pigment epithelium and pigment depositions in both eyes (G,H). Choroidal thickness at the fovea and 1500 µm nasal to the fovea measured 284 µm and 405 µm in the right eye and 295 µm and 448 µm in the left eye, respectively.

**Figure 3:**
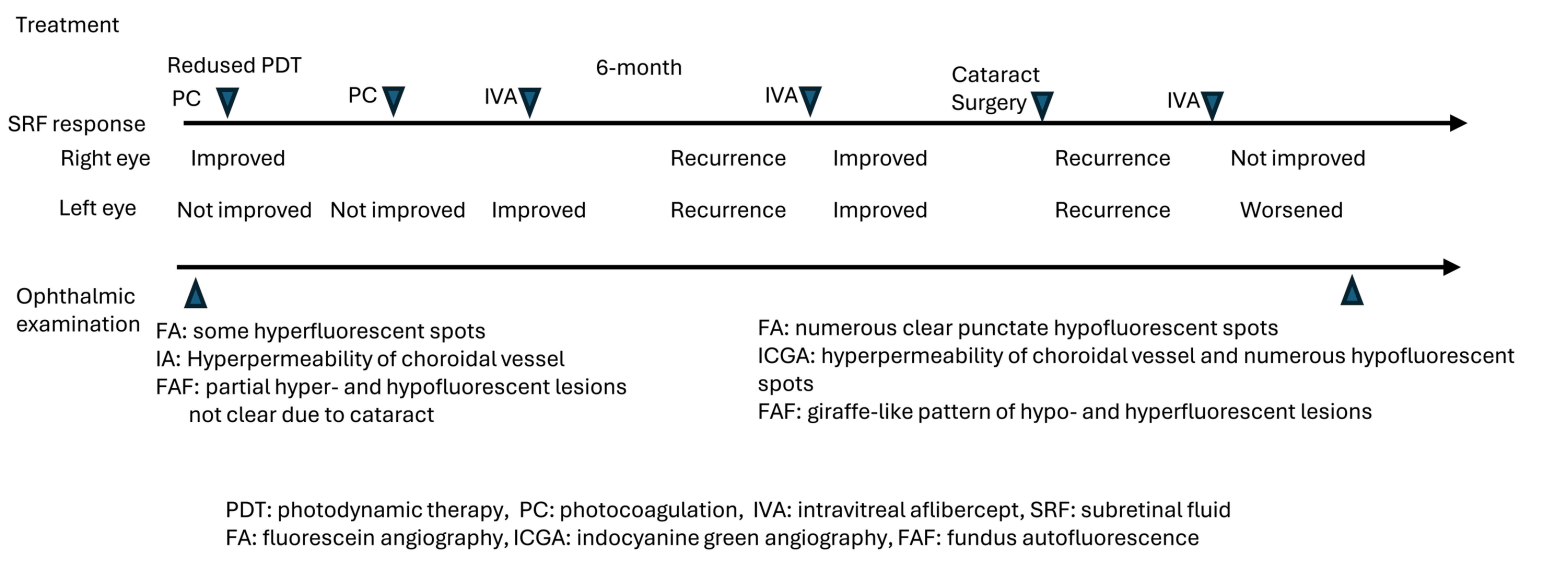
Timeline of treatment and imaging findings

The patient subsequently complained of blurred vision in both eyes due to cataract progression; therefore, bilateral cataract surgery was performed. Although BCVA improved in both eyes after the cataract surgery, both eyes again developed SRF one month after surgery. The recurrent SRF showed no response, even after the administration of additional IVA; therefore, additional angiography and FAF images were obtained. FA showed hyperfluorescence in the same areas as on the initial exam, as well as numerous clear punctate hypofluorescent spots in both eyes that were not observed at the initial first visit (Figure 2A,B). ICGA, as well as FA, showed numerous hypofluorescent spots in both eyes as well as the same choroidal vascular hyperpermeability seen on the initial examination (Figure 2C,D). FAF images showed hyperfluorescence consistent with the area of hypofluorescence on both FA and ICGA, a characteristic giraffe-like pattern of hypo- and hyperfluorescent lesions (Figure 2E,F). OCT well-depicted SRF with irregular retinal pigment epithelium (RPE) and pigment depositions (Figure 2G,H). The timeline of treatment and imaging findings are shown in Figure 3.

**Figure 4:**
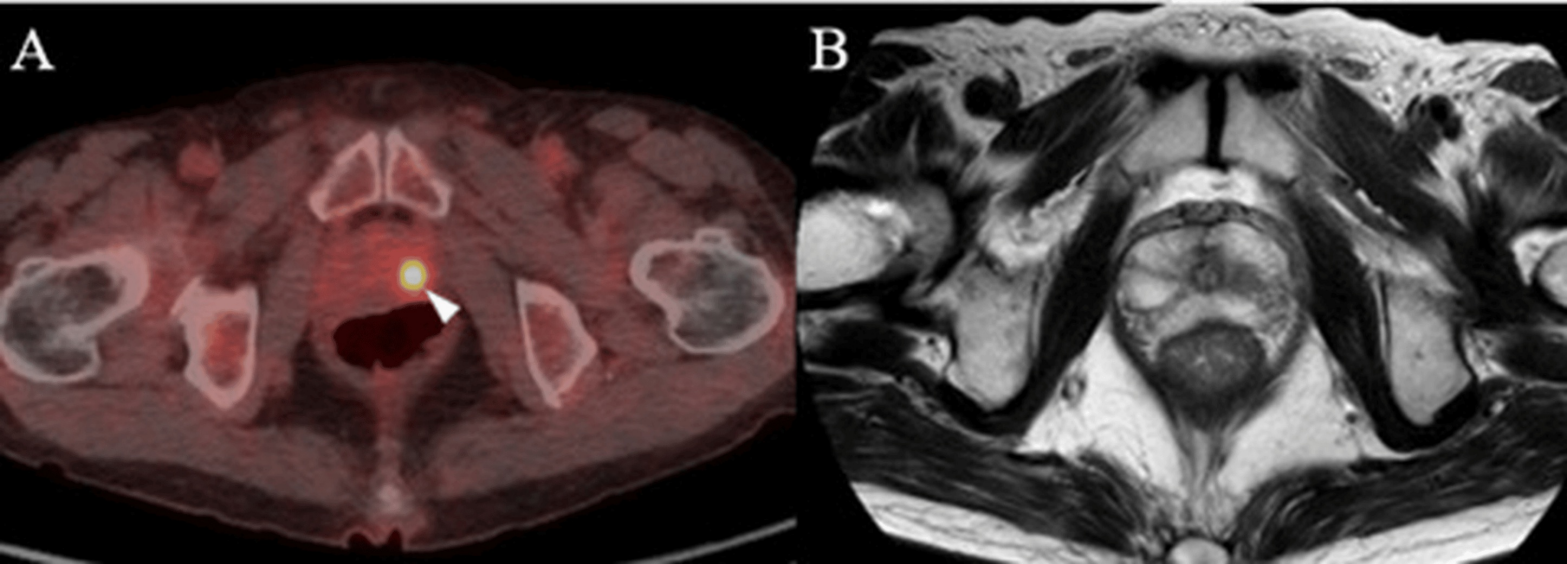
The images of prostate cancer 2-Deoxygenated-2-fluoro-D-glucose positron emission tomography-computed tomography of the whole body showed a hyperintense lesion in the left lobe of the prostate (A), while magnetic resonance imaging showed a hypointense lesion in the same area (B). Prostate biopsy revealed a diagnosis of prostate cancer.

Based on these findings, the patient received a revised diagnosis of BDUMP. Subsequent systemic evaluation, including 2-deoxygenated-2-fluoro-D-glucose positron emission tomography-computed tomography and magnetic resonance imaging, revealed a suspicious lesion in the left lobe of the prostate. Serum testing subsequently demonstrated an elevated prostate-specific antigen (PSA) level of 11.96 ng/mL, leading to the diagnosis of prostate cancer (Figure 4A,B).

## Discussion

Klemp et al. [3] reported that BDUMP is primarily associated with endocrine carcinomas - 71% of female patients with BDUMP were diagnosed with urogenital carcinoma, while 50% of male patients with BDUMP were diagnosed with lung carcinoma. They also indicated that the primary malignancies were discovered at the same time or after the onset of BDUMP in 44% of cases, which contributes to the generally poor prognosis among these patients [3]. These findings emphasize the critical need for comprehensive systemic evaluations and malignancy screenings in patients diagnosed with BDUMP, even in the absence of a history of cancer. We have described herein such a case, in which BDUMP was diagnosed during the treatment of SRF with PPS, followed by a subsequent diagnosis of prostate cancer.

PPS, recently proposed as part of the pachychoroid disease spectrum, is a syndrome including pachychoroid features around the optic nerve as well as intra- and/or subretinal fluid [8,9]. The efficacy of treatment for PPS remains controversial. PDT has been reported to be effective [10,11]; however, some reports indicate that it is refractory to CSC [12]. Anti-vascular endothelial growth factor (VEGF) drugs are reported to be effective in some cases [13], but of limited benefit in others [8,14,15]. In our case, the patient showed an initial response to PDT and anti-VEGF therapy for PPS, which became refractory to SRF after he underwent cataract surgery. As no giraffe-like pattern was observed on his initial FAF images, the diagnosis of SRF due to PPS was made based on his history of steroid use, thick choroid, and dilated choroidal vessels. It should be noted that the presence of cataracts may have limited the sensitivity of autofluorescence imaging at the initial presentation, potentially obscuring subtle retinal pigment epithelial changes. Although it is not clear when BDUMP developed in this case, the SRF at the first visit was most likely caused by PPS. However, considering that SRF can be refractory due to PPS, the possibility that SRF was caused by BDUMP from the beginning cannot be ruled out despite the effectiveness of PPS treatment, given the presence of cataracts and the repeated recurrence of SRF. In retrospect, although the initial peripapillary pachychoroid features were compatible with PPS, the subsequent development of bilateral involvement accompanied by characteristic giraffe-like autofluorescence patterns and punctate hypofluorescent spots was more indicative of BDUMP than PPS. Therefore, close attention should be paid not only to color fundus imaging and OCT, but also to FAF imaging.

In the present case, SRF was initially attributed to PPS, while the progression of undiagnosed BDUMP gradually rendered the anti-VEGF injection therapy ineffective. The patient’s fundus was frequently monitored, and the fact that the characteristic findings of BDUMP were immediately detected but located only in the arcade vessels implied that the patient was in the early stages of BDUMP. 

Several cases have been reported of BDUMP being initially misdiagnosed and patients being treated for AMD; therefore, more attention should be paid to patients with retinal pigment epithelial irregularities. This also applies to cases of PPS with retinal pigment epithelial irregularities, as in the present case. In cases of refractory SRF, BDUMP should be considered in the differential diagnosis, and imaging findings, especially those of FAF, should be carefully monitored.

## Conclusions

In cases of treatment-resistant SRF, BDUMP should be considered in the differential diagnosis, even in patients without a history of malignancy or with features characteristic of the pachychoroid disease spectrum. Because BDUMP can masquerade as PPS or neovascular AMD, careful assessment of fundus imaging, including FAF, is important for distinguishing it from these conditions. Such cases highlight the importance of considering BDUMP and performing an appropriate systemic evaluation to identify a possible underlying malignancy.
